# Hoehn and Yahr Stage and Striatal Dat-SPECT Uptake Are Predictors of Parkinson’s Disease Motor Progression

**DOI:** 10.3389/fnins.2021.765765

**Published:** 2021-12-13

**Authors:** Holly Jackson, Judith Anzures-Cabrera, Kirsten I. Taylor, Gennaro Pagano, PASADENA Investigators, Claudia Altendorf, Prasinezumab Study Group, Markus Abt

**Affiliations:** ^1^Roche Products Ltd., Welwyn Garden City, United Kingdom; ^2^Department of Mathematics and Statistics, Lancaster University, Lancaster, United Kingdom; ^3^Roche Pharma Research and Early Development (pRED), Neuroscience and Rare Diseases Discovery and Translational Area, Roche Innovation Center Basel, Basel, Switzerland; ^4^Faculty of Psychology, University of Basel, Basel, Switzerland; ^5^Department of Basic and Clinical Neuroscience, IoPPN, King’s College London, London, United Kingdom

**Keywords:** PASADENA, PPMI (Parkinson’s Progression Markers Initiative), Parkinson’s disease, progression predictors, ridge regression, disease stage, Dat-SPECT imaging, MDS-UPDRS (Movement Disorder Society revision of Unified Parkinson’s Disease Rating Scale)

## Abstract

Currently, no treatments available for Parkinson’s disease (PD) can slow PD progression. At the early stage of the disease, only a subset of individuals with PD progress quickly, while the majority have a slowly progressive form of the disease. In developing treatments that aim to slow PD progression, clinical trials aim to include individuals who are likely to progress faster, such that a treatment effect, if one exists, can be identified easier and earlier. The aim of the present study was to identify baseline predictors of clinical progression in early PD. We analyzed 12-month data acquired from the PASADENA trial Part 1 (NCT03100149, *n* = 76 participants who were allocated to the placebo arm and did not start symptomatic therapy) and the Parkinson’s Progression Markers Initiative (PPMI) study (*n* = 139 demographically and clinically matched participants). By using ridge regression models including clinical characteristics, imaging, and non-imaging biomarkers, we found that Hoehn and Yahr stage and dopamine transporter single-photon emission computed tomography specific binding ratios (Dat-SPECT SBR) in putamen ipsilateral to the side of motor symptom onset predicted PD progression at the early stage of the disease. Further studies are needed to confirm the validity of these predictors to identify with high accuracy individuals with early PD with a faster progression phenotype.

## Introduction

Parkinson’s disease (PD) is the second most common neurodegenerative disorder (Nussbaum and Ellis, 2003). It is thought to affect roughly 1% of the population over 65 years old and 5% of the population over 85 years old (Wood-Kaczmar et al., 2006).

A reliable diagnostic test for PD is not yet available. The diagnosis of PD is often based on clinical symptoms. Current criteria for PD diagnosis include the presence of bradykinesia as well as resting tremor and/or rigidity (De Lau and Breteler, 2006). However, the gold standard for diagnosis is still pathological confirmation post-mortem (Belle et al., 2017). When diagnosis is investigated at autopsy, it is thought between 10 and 20% of previously clinically diagnosed cases were misdiagnosed (De Lau and Breteler, 2006). Even though clinical diagnosis of PD is based on motor symptoms, many individuals with PD also suffer from non-motor symptoms including dribbling, constipation and anxiety (Poewe, 2008).

Loss of dopaminergic neurons in the substantia nigra is considered the main cause of PD (Reeve et al., 2014), which is associated with loss of dopaminergic terminals in the forebrain, reduced dopamine release and thus, the onset of motor symptoms. The current gold standard of treatment for PD is Levodopa, which is converted to dopamine within the brain, which restores (at least partially) the levels of dopamine in the striatum (LeWitt, 2008). However, this effect is only transient, as it does not stop the progression of disease, as dopaminergic neurons continue to die. Neurodegeneration of dopaminergic neurons has been associated with the presence of aggregated alpha-synuclein in the form of Lewy bodies (Ruipérez et al., 2010).

Prasinezumab is a humanized immunoglobulin G1 monoclonal antibody designed to selectively bind aggregated alpha-synuclein at the C-terminus. PASADENA is a Phase II, randomized, double blind, placebo controlled trial that investigates the efficacy and safety of prasinezumab in early PD (Pagano et al., 2021). Here, the placebo arm from PASADENA was used to investigate the progression of the disease in individuals with early PD and to identify prognostic factors that can predict PD progression.

We aim to produce a model, to identify baseline characteristics, which can predict disease progression in individuals with PD. These predictors might also aid the identification of PD patients who are at risk of degenerating more rapidly. These patients are the ideal population to enroll into clinical trials to enable an earlier and faster detection of a treatment effect, if one exists, as, these patients may be more likely to respond to treatments aimed at slowing disease progression.

## Materials and Methods

### PASADENA Dataset

The PASADENA trial consisted of two parts: in Part 1 (first 52 weeks), participants were randomized to either placebo, prasinezumab 1,500 mg or prasinezumab 4,500 mg (3,500 mg for patients with body weight ≤ 65 kg; referred to as 4,500 mg group throughout) with a 1:1:1 allocation ratio; in Part 2 (second 52 weeks), participants randomized to treatment with prasinezumab in Part 1 remained on the same dose for the duration of Part 2, and those participants initially randomized to placebo were re-randomized to either 1,500 or 4,500 mg prasinezumab using a 1:1 allocation ratio. In the present study, placebo data from PASADENA Part 1 were analyzed.

The key inclusion criteria for PASADENA included: patients that were diagnosed with idiopathic PD with bradykinesia and one of rigidity or resting tremor and no other known or suspected cause of PD; patients aged between 40 and 80 years old; a visual evaluation of Dat-SPECT consistent with PD; a body weight in the range of 45–110 kg; a body mass index between 18 and 34 kg/m2; and either treatment naïve or on a stable dose of MAO-B inhibitor for at least 90 days (Pagano et al., 2021).

The PASADENA trial recruited 316 early stage (diagnosis within 2 years, Hoehn and Yahr Stage 1 or 2) dopamine-treatment naïve participants, 105 of which were assigned to the placebo arm, 105 to the prasinezumab 1,500 mg arm and 106 to the prasinezumab 4,500 mg arm. Although the protocol stated that during Part 1 of the trial participants should not start symptomatic treatment, there were 29 participants allocated to the placebo arm who did start symptomatic therapy during the first 52 weeks of the trial. For this reason, the models presented in this paper were created using the 76 dopamine-treatment naïve PD participants (training dataset) who remained naïve for the whole duration of Part 1 (52 weeks). The baseline characteristics of the dopamine-treatment naïve PD patients are shown in Table 1.

**TABLE 1:** Demographic and baseline characteristics of PASADENA and PPMI datasets.

Baseline characteristics	PASADENA (*n* = 76)	PPMI (*n* = 139)	*p*-value
Mean age, years (SD)	59.79 (8.52)	62.07 (8.52)	0.0623
Gender, male (%)	50 (65.79)	91 (65.47)	1.0000
Mean time since Diagnosis, months (SD)	9.55 (6.59)	6.97 (6.34)	0.0063
Hoehn and Yahr Stage (%)			
I	17 (22.37)	69 (49.64)	0.0002
II	59 (77.63)	70 (50.36)	
Mean MDS-UPDRS Part I (SD)	4.12 (2.96)	4.19 (3.02)	0.8588
Mean MDS-UPDRS Part II (SD)	4.99 (3.8)	4.74 (3.58)	0.6441
Mean MDS-UPDRS Part III (SD)	20.33 (8.42)	19.53 (8.65)	0.5125
Mean Total MDS-UPDRS (SD)	29.43 (10.97)	29.65 (11.69)	0.8943

*Two independent sample t-test used to test the difference in continuous variables and z-test used to test the differences in categorical variables.*

**FIGURE 1:**
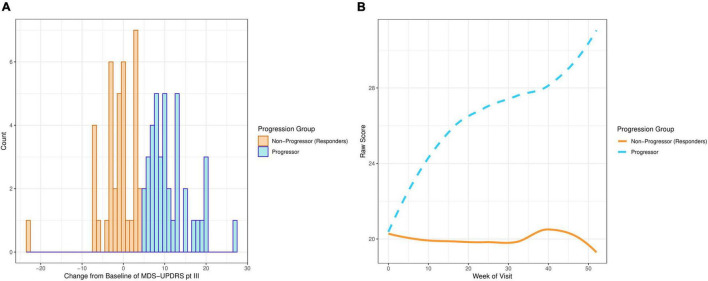
Plots showing MDS-UPDRS part III scores of the PD participants on the placebo arm in PASADENA. **(A)** Distribution of change from baseline. **(B)** Smoothed average score by week of visit.

The motor examination part of the Movement Disorder Society Unified Parkinson’s Disease Rating Scale (MDS-UPDRS part III) was used to define PD progression. Progression was defined as “at least a 5 point increase in MDS-UPDRS part III at week 52 in participants who have not started symptomatic treatment.” This cut-off score was chosen by PD experts using literature (Horváth et al., 2015). Figure 1A demonstrates that the distribution of the change from baseline at week 52 in MDS-UPDRS part III is bimodal, with one mode above a + 5 point change from baseline and the other mode below. Figure 1B shows average MDS-UPDRS part III data over 52 weeks using the “loess” smoothing function. This non-parametric function uses locally weighted regression to produce a fitted “line” that follows the densest area of the data, thus providing a graphical summary of the relationship between time and the PD patient’s MDS-UPDRS part III scores (Jacoby, 2000).

### Parkinson’s Progression Markers Initiative Dataset

PPMI is a multi-center observational study designed to identify PD progression biomarkers to improve understanding of the disease, and provide tools to increase the speed of therapeutic development. One of the commitments of PPMI is to make study data publicly available to the PD research community (Marek et al., 2011). The PD participants within this dataset are followed longitudinally. The data used in the present study were acquired from the LONI Image data archive in October 2020.^1^

PPMI data for the present analyses were selected such that the PPMI subsample was clinically and demographically comparable to the PASADENA placebo group (see Table 1). Initially, 396 PD patients were selected; however, after removing patients without follow-up data and restricting to patients with data around 12 months after baseline, the subset of PPMI dataset was reduced to 139 patients (test dataset) (see Table 1).

### Statistical Methods

Continuous measurements are reported with means and standard deviations. Binary variables are reported as proportions.

Four models were calculated to predict the progression of PD patients. These models used the baseline characteristics of the PD patients to predict if they would progress or not. The 76 PD participants from the placebo arm of PASADENA Part 1, were used to train these models. This training dataset included 39 progressors and 37 non-progressors. Furthermore, the 139 clinically matched PD patients from PPMI were used to validate these prediction models. This test dataset included 91 progressors and 48 non-progressors.

We followed two approaches to select the predictor variables to be included in the models: (1) using baseline data from the PASADENA trial by progression status, PD experts (G.P. and K.T.) were asked to select the variables that they considered to be the best predictors of progression based on their clinical expertise (clinical model); and (2) predictors for a “data-driven” model were selected by calculating the standardized mean differences for continuous variables, and odds ratios for binary ones. The variables selected by the PD experts were: baseline age, sex, MDS-UPDRS part III, Montreal cognitive assessment test (MoCA), REM sleep behavior disorder score, Dat-SPECT: Putamen-Ipsilateral and Dat-SPECT: Putamen-Contralateral. Baseline MDS-UPDRS part III was included as a covariate as it was part of the progressor definition.

**FIGURE 2:**
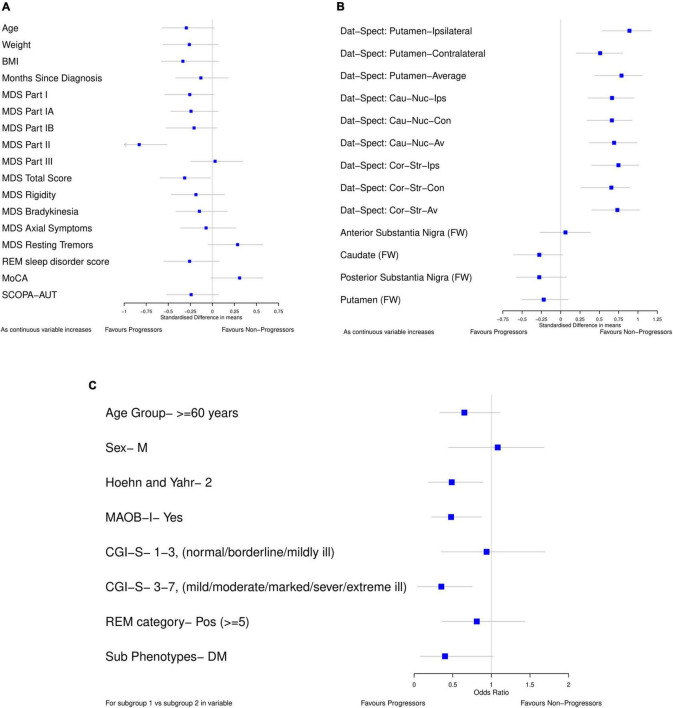
Forest plots showing difference in baseline characteristics between progressors and non-progressors in the placebo arm from PASADENA. **(A,B)** Standardized mean difference (80% confidence intervals). **(C)** Odds ratio (80% confidence intervals); for each variable, the reference group is listed. All plots were calculated using 1000 bootstrap samples.

For the data-driven model, standardized mean differences between progressors and non-progressors were calculated using bootstrap (Figures 2A,B) for all the continuous variables available in the PASADENA trial. Binary and ordinal variables were explored by calculating bootstrap samples of odds ratios between progressors and non-progressors (Figure 2C). The ordinal variable Clinical Global Impression of Severity and Improvement (CGI-S) was grouped as stages 1–3 vs. 4–7, and 1–2 vs. 3–7. Due to the training dataset being small (only 76 patients), the results from the standardized mean differences and odds ratios may not be reliable estimates for the whole population (Hackshaw, 2008), therefore bootstrapping was used to help combat this (Dwivedi et al., 2017). Bootstrapping is a re-sampling method, with replacement (Chernick and LaBudde, 2014). It allows the iterative re-sampling of the original data, to determine the standardized mean difference (and odds ratio) on the sample. An average standardized mean difference (and odds ratio) can then be found from the many bootstrap models calculated. We ran the “boot” function from the “boot” package (Canty and Boot, 2020) in RStudio (RStudio Team, 2020) 1,000 times to find the average standardized mean difference (and odds ratio).

Forest plots (Figure 2) were used to select the variables for the data-driven model which clearly differentiated between progressors and non-progressors. All variables in which 80% confidence intervals did not contain the null value (and were not correlated) and the variable whose confidence interval crossed the null value by the smallest amount [MoCA, SMD 0.308 (80% CI –0.019, 0.575)] were chosen. The selected variables for the data driven model were: Dat-SPECT: Putamen-Ipsilateral, Hoehn and Yahr Stage, if MAOB-I had been taken from baseline, MoCA and MDS-UPDRS part II. In addition, all the models include baseline MDS-UPDRS part III as a covariate to adjust for baseline differences.

All the Dat-SPECT variables in different regions of the brain showed a good separation between progressors and non-progressors. However, as they are highly correlated, we chose the DaT-SPECT variable with the largest standardized mean difference: Dat-SPECT: Putamen-Ipsilateral. Additionally, CGI-S was left out of the model as the majority of patients (53) were in the mildly ill group and there were only 3, 5, and 15 patients in the normal, borderline ill and moderately ill groups, respectively.

Missing data was low in the training dataset, only one participant had missing MoCA at baseline. However, the test dataset had a higher proportion of missing data: 36 participants did not have values recorded for either Dat-SPECT Putamen-Ipsilateral or Dat-SPECT Putamen-Contralateral, and an additional participant did not have an REM sleep behavior disorder score recorded. We used the “missForest” function to estimate these missing values in both datasets (Stekhoven, 2015). This is a non-parametric function, which uses the random forest algorithm to iteratively impute missing values and it makes few assumptions about the structure of the data (Stekhoven and Bühlmann, 2012). All 11 predictor variables were used (along with the month of visit for the test dataset), and the progression group was labeled as the response variable in the “missForest” function.

Ridge logistic regression was used to create a model that predicts progression in participants after 12 months of follow-up. The aim of the model was to separate progressors from non-progressors using different prognostic factors.

Ridge regression deals with the issue of collinearity in regression methods, without eliminating variables (McDonald, 2009). It allows the inclusion of collinear predictor variables, due to the penalty it introduces on the size of regression coefficients to enable shrinkage. This method reduces the size of the coefficients estimates (shrinking them toward zero) guaranteeing that no variables are eliminated from the model and making it more accurate for the extreme values of the predictor variables (Hastie et al., 2008). Due to the penalty on the coefficients, they have a size constraint, however, this constraint will depend on the magnitude of the predictor variables in the model. Therefore, so that each coefficient has the same size constraint, the training dataset must first be standardized (Hastie et al., 2008). The “preProcess” function in the “caret” package (Kuhn et al., 2020) in RStudio (RStudio Team, 2020) was used to center and scale both the training and test datasets. The ridge regression model was created using the “glmnet (alpha = 0)” function from the “glmnet” package (Friedman et al., 2010). As the training dataset was small (*n* = 76), the regression models may not be representative of the whole population (Hackshaw, 2008), therefore bootstrapping was used to help combat this issue (Dwivedi et al., 2017). It allowed the iterative re-sampling of the original data and recalculation of the ridge regression model on each sample. An average model can then be found from the many bootstrap models calculated. We run the “boot” function from the “boot” package (Canty and Boot, 2020) in RStudio (RStudio Team, 2020) 1,000 times to find the average coefficients from the ridge regression model. We then used these averaged coefficients as the coefficients in our prediction models. All analyses were performed in R using RStudio (RStudio Team, 2020).

For both the clinical and data-driven models, the importance of each predictor was then inspected using their variable importance calculated using the random forest algorithm (Liaw and Wiener, 2002). Each classification tree in the random forest is grown on a sample of the data using all predictor variables in the model, the classification tree is then tested on all the data excluding the sample. One predictor variable is then permuted and the classification tree is tested again on all the data not in the sample. The difference between the accuracy of the tree on the data before and after the variable permutation, is the decrease in accuracy for that specific predictor variable. This is then averaged for each classification tree in the random forest. This mean difference is the permutation importance of the predictor variable (Archer and Kimes, 2008). This process is repeated for all variables in the model. The variables with larger importance are more likely to be relevant predictors of progression. This permuted importance measure was used to rank the true predictive value of each variable. New prediction models were inspected, which included the more predictive variables. These models were compared and the one with the largest predictive accuracy was chosen as the second model.

The Non-Progressor Predictive Value (NPV), Progressor Predictive Value (PPV), Total Predictive Value (TPV), Sensitivity and Specificity were used to measure the predictive accuracy of the models. The NPV is the proportion of predicted non-progressors who actually do not progress (Parikh et al., 2008). The PPV is the proportion of predicted progressors who actually progress (Parikh et al., 2008). The TPV is the proportion of total predictions the model gets correct. Sensitivity is the proportion of patients who actually progress that the model predicts correctly and the specificity is the proportion of patients who do not progress, which the model predicts correctly (Parikh et al., 2008). The Brier score was also used to measure the performance of the prediction models. It is the average squared difference between the actual outcome of each patient in the test dataset (0 for non-progressors and 1 for progressors) and the predicted probability of them progressing (Benedetti, 2010). Hence, the smallest and most beneficial Brier score is equal to 0.

Additionally, the receiver operating characteristic (ROC) curve was calculated for all models, as was the area under the curve (AUC). The ROC curve involves plotting the true positive rate (sensitivity) against the false-positive rate (1-specificity). Furthermore, the AUC represents the probability that a progressor chosen at random, is rated more likely to progress than a non-progressor, chosen also at random (Lora et al., 2016). Thus, the largest and most beneficial AUC is equal to 1.

Other methods partnered with bootstrap sampling were tested including LASSO regression, logistic regression using maximum likelihood estimation and random forests. However, for the models explored, ridge regression had the largest predictive accuracy.

We investigated two different ridge logistic regression models:

1. Clinically selected model, where the predictor variables were selected by PD experts.
2. Data-driven model, here the data from the PASADENA trial was used to select the predictor variables which differentiated between progressors and non-progressors.

## Results

Demographic and baseline characteristics of the 76 PD participants in the PASADENA trial (training dataset) and the 139 clinically matched PD participants in the PPMI cohort (test dataset) are presented in Table 1. The *t*-test was used to investigate the difference in means of the continuous variables and the *z*-test was used to investigate the difference in proportions for the two binary variables. The *p*-values of these tests are shown in Table 1. The two datasets had a similar: mean age, proportion of men and mean MDS-UPDRS Part I, II, III and total scores. They did, however, differ in the time from PD diagnosis (*p* = 0.0063) and the Hoehn and Yahr stage (*p* = 0.0002). PASADENA had a larger mean time since PD diagnosis (9.55 months), compared to PPMI (6.97 months) and it had a larger proportion of Hoehn and Yahr stage II (77.63%), than the PPMI database (50.36%). This could affect the accuracy of the Hoehn and Yahr Stage as a predictor of progression.

The clinically selected model 1, which includes variables considered by PD experts to be the best predictors, is displayed in Table 2. As expected, age had a positive coefficient, with older patients declining faster than younger patients. In addition, both Dat-SPECT variables had negative coefficients, with lower Dat-SPECT values (i.e., more neurodegeneration) associated with fast progression and hence they are more likely to progress. The predictive values of each predictor are displayed in Table 2, which indicates that Ipsilateral putamen Dat-SPECT SBR was the most important variable in predicting the progression of PD patients.

**TABLE 2:** Clinically selected model 1 to predict motor progression in individuals with PD.

Variables	Beta	SE	Exp(Beta)	Permutation
				importance
Intercept	0.066	0.260	1.068	–
Age	0.064	0.203	1.066	–0.0007
Sex	0.026	0.199	1.026	–0.0068
Ipsilateral putamen Dat-SPECT SBR	–0.692	0.373	0.501	0.0175
Contralateral putamen Dat-SPECT SBR	–0.046	0.269	0.955	–0.0093
REM sleep behavior score	0.275	0.234	1.317	–0.0012
MoCA	–0.224	0.211	0.799	–0.0001

*Beta, standardized bootstrapped coefficients; SE, standard error, estimated by the standard deviation of the bootstrapped beta coefficients; Exp(Beta), exponential of the standardized bootstrapped coefficients; the model also included MDS-UPDRS Part III as a covariate.*

Multiple clinical models were considered, focusing on the variables which were ranked most important and MDS-UPDRS part III as a covariate. The model which performed best, clinically selected model 2, is shown in Table 3. Out of the models explored it produced the largest NPV, PPV and TPV.

**TABLE 3:** Clinically selected model 2 to predict motor progression in individuals with PD.

Variables	Beta	SE	Exp(Beta)
Intercept	0.062	0.253	1.064
Age	0.047	0.198	1.048
Ipsilateral putamen Dat-SPECT SBR	–0.737	0.312	0.479
MoCA	–0.210	0.218	0.811

*Beta, standardized bootstrapped coefficients; SE, standard error, estimated by the standard deviation of the bootstrapped beta coefficients; Exp(Beta), exponential of the standardized bootstrapped coefficients; the model also included MDS-UPDRS Part III as a covariate.*

The first data driven model is displayed in Table 4. As expected, Hoehn and Yahr had a positive coefficient, with higher stages associated with fast progression. Again, Dat-SPECT SBRs in the ipsilateral putamen had a negative coefficient. MDS-UPDRS part II had a positive coefficient, with higher scores associated with fast progression.

**TABLE 4:** Data Driven model 1 to predict motor progression in individuals with PD.

Variables	Beta	SE	Exp(Beta)	Permutation
				importance
Intercept	0.093	0.273	1.097	–
Ipsilateral putamen Dat-SPECT SBR	–0.833	0.248	0.435	0.0243
Hoehn and Yahr	0.224	0.267	1.251	0.0070
MAOB-I taken at baseline	0.284	0.225	1.328	0.0065
MoCA	–0.178	0.222	0.837	–0.0019
MDS-UPDRS Part II	0.835	0.287	2.305	0.0427

*Beta, standardized bootstrapped coefficients; SE, standard error, estimated by the standard deviation of the bootstrapped beta coefficients; Exp(Beta), exponential of the standardized bootstrapped coefficients; the model also included MDS-UPDRS Part III as a covariate.*

The predictive accuracy of the variables within the data-driven model were further compared using the permutation importance of each variable. Their variable importance is also displayed in Table 4, which shows the two most important variables were MDS-UPDRS part II and Ipsilateral putamen Dat-SPECT. Several models were investigated, focusing on the variables which were ranked most important including MDS-UPDRS part III. The model which performed best in terms of NPV, PPV and TPV, the second data driven model, is shown in Table 5.

**TABLE 5:** Data driven model 2 to predict motor progression in individuals with PD.

Variables	Beta	SE	Exp(Beta)
Intercept	0.05	0.253	1.051
Ipsilateral putamen Dat-SPECT SBR	–0.785	0.295	0.456
Hoehn and Yahr	0.229	0.252	1.257

*Beta, standardized bootstrapped coefficients; SE, standard error, estimated by the standard deviation of the bootstrapped beta coefficients; Exp(Beta), exponential of the standardized bootstrapped coefficients; the model also included MDS-UPDRS Part III as a covariate.*

**FIGURE 3:**
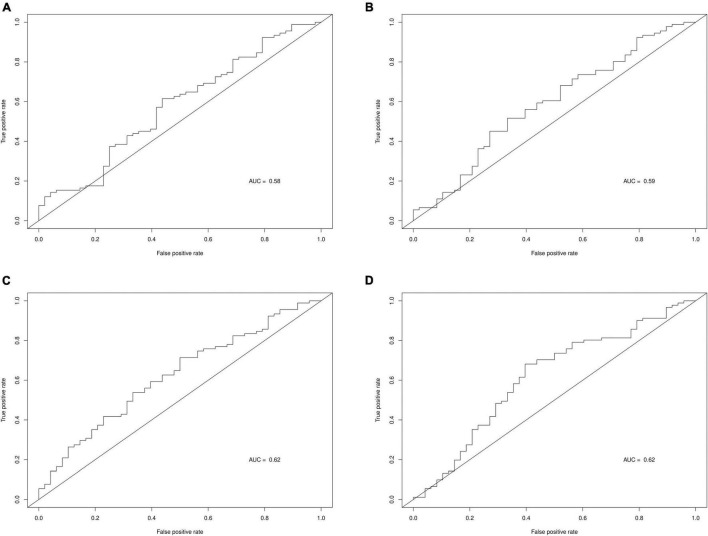
ROC curves and AUC for each prediction model. **(A)** Clinically selected prediction model 1. **(B)** Clinically selected prediction model 2. **(C)** Data driven prediction model 1. **(D)** Data driven prediction model 2.

Table 6 displays the predictive accuracy of the two clinically selected models and the two data driven models, in predicting the progression of the 139 PD participants in the PPMI dataset. Table 6 shows the second data driven model had a high predictive accuracy, producing the joint largest NPV = 0.45 and the largest PPV = 0.75. However, the second clinically selected model also had a high predictive accuracy. It produced the joint largest NPV = 0.45 and largest TPV = 0.62 and sensitivity = 0.71. The first data driven model produced the largest specificity = 0.67. Both the clinically selected models had very similar small Brier scores, 0.2395 vs. 0.2363. Figure 3 displays the ROC curve and AUC for each model, where the two data driven models gave the joint largest AUC.

**TABLE 6:** Predictive accuracy of models.

Model	NPV	PPV	TPV	Sensitivity	Specificity	Brier score
Clinically selected 1	0.40	0.68	0.59	0.69	0.40	0.2395
Clinically selected 2	0.45	0.71	0.62	0.71	0.44	0.2363
Data driven 1	0.42	0.74	0.56	0.51	0.67	0.2752
Data driven 2	0.45	0.75	0.60	0.59	0.63	0.2497

## Discussion

Our finding suggests that Hoehn and Yahr stage and Ipsilateral putamen Dat-SPECT predict progression of motor signs in early PD. These results were derived by using four models to predict PD progression at 12 months evaluating a sample of 76 individuals with early PD (training set from the PASADENA placebo arm) and validating them in a sample of 139 clinically matched PD individuals (validation set from the PPMI database).

Data driven models gave a larger specificity than the clinically selected models, due to correctly predicting a larger proportion of the actual non-progressors, 0.67 and 0.63 vs. 0.40 and 0.44. However, the data driven models generated a lower sensitivity, as they also incorrectly predicted large quantities of progressors, 0.51 and 0.59 vs. 0.69 and 0.71. All 4 models produced quite similar non-progressor predictive values (predicted non-progressors who will actually not progress), 40%, 45, 42, and 45%. Furthermore, the data driven models had larger progressor predictive values (predicted progressors who will actually progress), 74 and 75% vs. 68 and 71%. Additionally, the two clinically selected models generated better (lower) Brier scores than the data driven models: 0.2395 and 0.2363 vs. 0.2752 and 0.2497. Lastly, the two data driven models produced larger AUC than the two clinically selected models: 0.62 and 0.62 vs. 0.58 and 0.59.

The predictive strength of each model depends on the relative power of each predictor variable. However, the second clinically selected model and both data driven models performed well for different measures of predictive accuracy. The second clinically selected model yielded the joint largest NPV, largest sensitivity, largest TPV and the lowest Brier score. However, the first data driven model gave the largest specificity and joint highest AUC and the second data driven model produced the joint best NPV, the best PPV and joint highest AUC. To decide which model performed best, we must choose which prediction measure we believe to be the most important. In this situation we found the mean of the PPV, NPV, and TPV for each model, which produced 0.56, 0.59, 0.57, and 0.6, respectively. The second data driven model appears to be the most well rounded model taking into account these three predictive values, and indeed when sensitivity, specificity and AUC are also included, the second data driven model still produced the largest mean of these 6 performance measures. Therefore, we conclude the second data driven model, which included the variables: Hoehn and Yahr stage, Ipsilateral putamen Dat-SPECT and MDS-UPDRS Part III as a covariate, is the optimal prediction model.

We note that there may be other baseline variables which have an effect on the progression of PD patients, which have not been explored. Another limitation of the models, is the small PASADENA dataset used to train said models and the differences between the training and test datasets. The two datasets had differing mean time from diagnosis and proportions of PD patients in Hoehn and Yahr Stage I and II. Furthermore, the follow-up time did not match between the two datasets. In PASADENA, progression was defined 52 weeks after baseline, whereas in PPMI, progression was defined at a patient’s visit closest to 12 months after baseline, where the visit could have been anywhere between 10 and 14 months after baseline. These differences could also affect the predictive accuracy of our models and levels of validation.

Finally, there were missing values in both the training and test datasets. The one missing value in the training dataset will not have a large effect on the accuracy of the prediction models. However, the 36 patients of the 139 in the PPMI dataset (25.9%) who did not have an Ipsilateral putamen or Contralateral putamen Dat-SPECT value could have affected the accuracy of our prediction models if the missing values had not been imputed correctly.

Further studies are needed to validate these results in an independent dataset and evaluate other baseline characteristics not investigated here. In addition, these prediction models could be further explored by investigating different cut off values for determining the probability of becoming a progressor or not. Here, a cut-off of 0.5 was used, thus, any patient with a predicted probability of progressing above 0.5, was predicted to progress. However, other cut-off values could be considered toward improving the above model’s predictive accuracy.

## Conclusion

In conclusion, four models predicting motor sign progression in early PD patients are presented. They were trained on a sample of 76 PD patients who had not started symptomatic dopaminergic therapy assigned to the placebo arm of the phase II PASADENA study. These models were validated in 139 demographically and clinically matched PD patients from the PPMI database. Baseline Hoehn and Yahr stage and Ipsilateral putamen Dat-SPECT SBR best predicted motor sign progression in early PD. Further studies are needed to confirm the validity of these predictors to identify individuals with early PD with faster progression phenotype.

## Data Availability Statement

Qualified researchers may request access to individual patient-level data through the clinical study data request platform (https://vivli.org/). Further details on Roche’s criteria for eligible studies are available at https://vivli.org/members/ourmembers/. For further details on Roche’s Global Policy on the Sharing of Clinical Information and how to request access to related clinical study documents, see https://www.roche.com/research_and_development/who_we_are_how_we_work/clinical_trials/our_commitment_to_data_sharing.htm. Data used in the preparation of this article were obtained from the Parkinson’s Progression Markers Initiative (PPMI) database (www.ppmi-info.org/access-data-specimens/download-data). For up-to-date information on the study, visit ppmi-info.org. Further inquiries can be directed to the corresponding author/s.

## Ethics Statement

Participants were identified for potential recruitment using site-specific recruitment plans prior to consenting to take part in this study. Recruitment materials for participants had received Institutional Review Board or Ethics Committee approval prior to use. The following Institutional Review Boards ruled on ethics of the PASADENA study: Ethikkommission der Medizinischen Universität Innnsbruck, Innsbruck, Austria; Comité de Protection des Personnes (CPP) Ouest IV, Nantes, France; Ethikkommission der Universität Leipzig and Geschäftsstelle der Ethikkommission an der medizinischen Fakultät der Universität Leipzig, Leipzig, Germany; Ethikkommission der Fakultät für Medizin der Technischen Universität München, München, Germany; Ethikkommission der Universität Ulm (Oberer Eselsberg), Ulm, Germany; Landesamt für Gesundheit und Soziales Berlin and Geschäftsstelle der Ethik-Kommission des Landes Berlin, Berlin, Germany; Ethikkommission des FB Medizin der Philipps-Universität Marburg, Marburg, Germany; Ethikkommission an der Medizinischen Fakultät der Eberhard-Karls-Universität und am Universitätsklinikum Tübingen, Tübingen, Germany; Ethikkommission an der Med. Fakultät der HHU Düsseldorf, Düsseldorf, Germany; Ethikkommission der LÄK Hessen, Frankfurt, Germany; CEIm Hospital Universitari Vall d’Hebron, Barcelona, Spain; Copernicus Group Independent Review Board, Puyallup, WA, United States; Western Institutional Review Board, Puyallup, WA, United States; The University of Kansas Medical Center Human Research Protection Program, Kansas City, KS, United States; Oregon Health & Science University Independent Review Board, Portland, OR, United States; Northwestern University Institutional Review Board, Chicago, IL, United States; Spectrum Health Human Research Protection Program, Grand Rapids, MI, United States; The University of Vermont Committees on Human Subjects, Burlington, VT, United States; Beth Israel Deaconess Medical Center Committee on Clinical Investigations, New Procedures and New Forms of Therapy, Boston, MA, United States; Vanderbilt Human Research Protection Program Health, Boston, MA, United States; Vanderbilt Human Research Protection Program Health, Nashville, TN, United States; University of Maryland, Baltimore Institutional Review Board, Baltimore, MD, United States; University of Southern California Institutional Review Board, Los Angeles, CA, United States; Columbia University Medical Center Institutional Review Board, New York, NY, United States; University of Southern California San Francisco Institutional Review Board, San Francisco, CA, United States; University of Pennsylvania Institutional Review Board, Philadelphia, PA, United States; and HCA – HealthOne Institutional Review Board, Denver, CO, United States. All Institutional Review Boards gave ethical approval of the study.

## Author Contributions

GP, KT, and JA-C designed the study. HJ and JA-C were involved in data collection and analyzing the data. All authors contributed to data interpretation, revised and gave input on the article, and approved the final manuscript.

## Conflict of Interest

HJ was a paid intern of F. Hoffmann-La Roche Ltd. JA-C, KT, and GP were full-time employees and shareholders of F. Hoffmann-La Roche Ltd.

## Publisher’s Note

All claims expressed in this article are solely those of the authors and do not necessarily represent those of their affiliated organizations, or those of the publisher, the editors and the reviewers. Any product that may be evaluated in this article, or claim that may be made by its manufacturer, is not guaranteed or endorsed by the publisher.

## Footnotes

1
https://ida.loni.usc.edu/login.jsp
